# Incorporation of molecular characteristics into endometrial cancer management

**DOI:** 10.1111/his.14015

**Published:** 2019-12-17

**Authors:** Lisa Vermij, Vincent Smit, Remi Nout, Tjalling Bosse

**Affiliations:** ^1^ Department of Pathology Leiden University Medical Center Leiden the Netherlands; ^2^ Department of Radiation Oncology Leiden University Medical Center Leiden the Netherlands

**Keywords:** adjuvant treatment, endometrial carcinoma, lymphovascular space invasion, mismatch repair, molecular classification, p53, *POLE*, risk stratification

## Abstract

Histopathological evaluation including subtyping and grading is the current cornerstone for endometrial cancer (EC) classification. This provides clinicians with prognostic information and input for further treatment recommendations. Nonetheless, patients with histologically similar ECs may have very different outcomes, notably in patients with high‐grade endometrial carcinomas. For endometrial cancer, four molecular subgroups have undergone extensive studies in recent years: *POLE* ultramutated (*POLE*mut), mismatch repair‐deficient (MMRd), p53 mutant (p53abn) and those EC lacking any of these alterations, referred to as NSMP (non‐specific molecular profile). Several large studies confirm the prognostic relevance of these molecular subgroups. However, this ‘histomolecular’ approach has so far not been implemented in clinical routine. The ongoing PORTEC4a trial is the first clinical setting in which the added value of integrating molecular parameters in adjuvant treatment decisions will be determined. For diagnostics, the incorporation of the molecular parameters in EC classification will add a level of objectivity which will yield biologically more homogeneous subclasses. Here we illustrate how the management of individual EC patients may be impacted when applying the molecular EC classification. We describe our current approach to the integrated diagnoses of EC with a focus on scenarios with conflicting morphological and molecular findings. We also address several pitfalls accompanying the diagnostic implementation of molecular EC classification and give practical suggestions for diagnostic scenarios.

## Introduction

In the 4th edition of the *World Health Organisation (WHO) classification of tumours of female reproductive organs*,^1^ the definition for endometrial cancer (EC) entities were mainly based on histological characteristics supplemented with immunohistochemical markers. These microscopy‐based diagnoses are the current standard and serve as important input for (adjuvant) treatment decisions. However, considerable interobserver variation, in particular in high‐grade EC, is recognised, and centralised review prior to trial inclusion has pointed out that the therapeutic consequences are not negligible.^2^, ^3^ This situation prompted research into the incorporation of robust diagnostic markers, which yields novel narrowly defined diagnostic entities.

**Figure 1:**
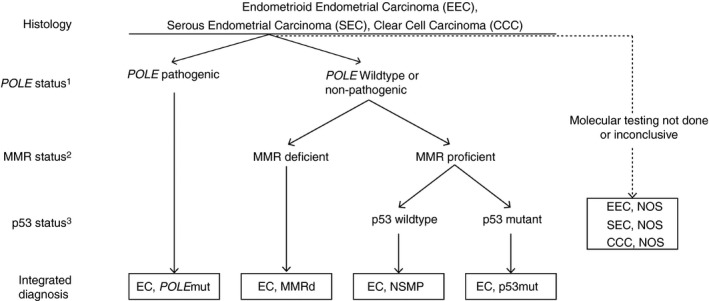
Diagnostic algorithm for a ‘histomolecular’ endometrial cancer classification. ^1^Pathogenic polymerase‐epsilon (*POLE*) variants include: P286R, V411L, S297F, A456P and S459F. ^2^Mismatch repair protein (MMR) deficiency is defined by the loss of one or more MMR‐proteins (MLH1, PMS2, MSH2 and MSH6). ^3^p53 immunohistochemistry (IHC) is an acceptable surrogate marker for *TP53* mutational status in MMR‐proficient, *POLE* wild‐type endometrial cancer (EC).^50^

During the last decade a paradigm shift was invoked when the results from The Cancer Genome Atlas (TCGA) project were published.^4^ The TCGA elegantly showed the molecular diversity of EC in which four distinct molecular subgroups were recognised based on somatic copy number alterations (SCNA) and tumour mutational burden. These four subgroups include: (i) ultra‐mutated ECs characterised by pathogenic variants in the exonuclease domain of DNA polymerase‐epsilon (*POLE*); (ii) hyper‐mutated ECs characterised by microsatellite instability (MSI); (iii) a copy‐number low subgroup with a low mutational burden; and (iv) a copy‐number high group with frequent *TP53* mutations.^4^ Subsequent studies showed and validated the prognostic relevance of this molecular stratification by using surrogate markers that enable the identification of EC subgroups analogous to the four described by the TCGA.^5^, ^6^ This has formed the basis of restructuring EC classification, using only a few key molecular aberrations, into *POLE* ultramutated (*POLE*mut) EC, MMRd EC, NSMP EC and p53abn EC (Figure 1). These novel integrated ‘histomolecular’ diagnostic entities not only provide more accurate prognostic information, they also provide an opportunity to improve the current clinical management for EC patients. This novel diagnostic algorithm has shown promise in refining adjuvant treatment recommendations (radio‐ and or chemotherapy), which currently does not incorporate molecular characteristics.

Adjuvant treatment is currently recommended based on a patient's individual risk (low‐, intermediate‐ and high‐risk) comprised of a combination of clinical (age) and pathological (FIGO stage, tumour type, grade and the presence of unequivocal lymphovascular space invasion (LVSI) factors.^7^, ^8^, ^9^, ^10^, ^11^, ^12^, ^13^, ^14^ How the additional molecular information should be incorporated into this risk‐based approach has still to be determined. It seems prudent, however, that treatment de‐escalation is considered in EC with favourable molecular factors (e.g. *POLE*mut EC) and intensified treatments are considered in the presence of unfavourable factors (e.g. p53mut EC). The ongoing PORTEC4a trial (http://ClinicalTrials.gov Identifier: NCT03469674) is the first clinical trial to prospectively investigate the incorporation of molecular EC characteristics in the context of patients who would currently be classified as high–intermediate risk based on clinicopathological factors alone. Standard postoperative vaginal brachytherapy (VBT) is being compared to adjuvant treatment based on an individual's molecular‐integrated risk profile, including no adjuvant treatment, VBT or external beam radiotherapy (EBRT). This randomised trial will provide essential information on how the ‘histomolecular’ entities may be used to personalise adjuvant treatment by decreasing both over‐ and undertreatment.

In this review, we illustrate clinical scenarios in which integration of the molecular characteristics into the current clinicopathological classification may shift the adjuvant treatment recommendations. We also discuss how the integrated molecular entities may provide clues to specific targeted treatment modalities that can be exploited in advanced‐stage or recurrent disease. For a more detailed background on the molecular EC classification, as well as assays that may be used for the diagnosis, the reader is referred to other reviews.^15^, ^16^, ^17^

## *POLE*mut high‐grade endometrial carcinoma

**Figure 2:**
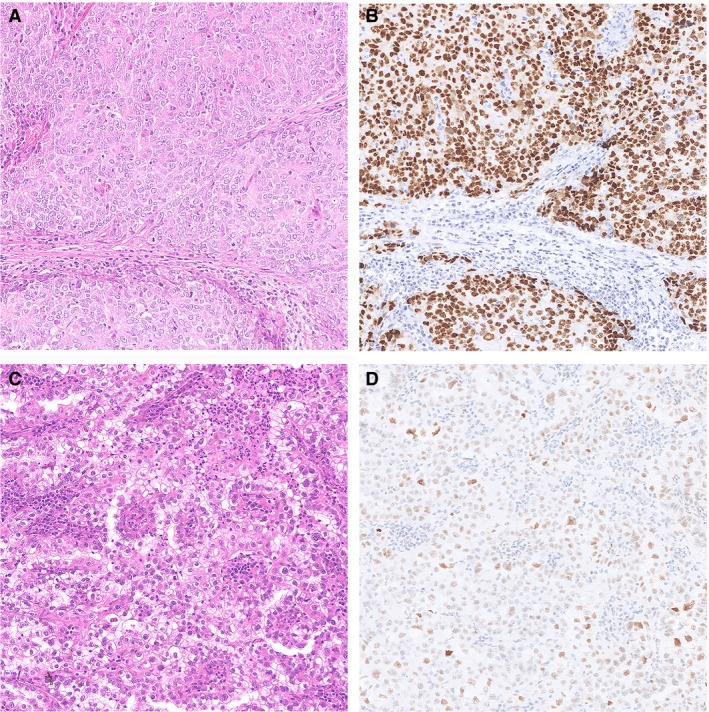
Two examples of polymerase‐epsilon mutated endometrial cancer (EC) with a pathogenic polymerase‐epsilon ultramutated (*POLE*mut) EC. **A**,**B**, Haematoxylin and eosin (H&E) stain of an EC diagnosed as endometrioid EC (FIGO Grade 3), based on solid growth, with aberrant mutant‐like p53 immunostaining. Molecular profiling showed the presence of a *POLE* P286R variant and a *TP53* mutation. **C**,**D**, H&E of an EC case originally diagnosed as mixed endometroid and clear cell EC with scattered nuclear p53 immunostaining interpreted as wild‐type p53. Molecular profiling showed a *POLE* V411L variant and no mutations in *TP53*.

Pathogenic *POLE* variants in the exonuclease domain of the *POLE* gene comprise approximately 10% 6of all endometrioid EC (EEC),^4^ and in the majority consists of one of the five hot‐spots: P286R, V411L, S297F, A456P and S459F. In the molecular EC classification these cases are referred to as ‘*POLE*ultramutated’ or ‘*POLE*mut EC’. Typical *POLE*mut EC features include: presentation at relatively young age and early stage, high tumour grade with scattered tumour giant cells and a prominent lymphocytic infiltrate.^4^, ^18^ Although *POLE*mut EC are mainly microsatellite‐stable (MSS), unstable cases with MMR‐protein loss have been described.^4^ Importantly, the occurrence of secondary *TP53* mutations have been reported in up to 42% of *POLE*mut EC, sometimes resulting in subclonal mutant‐like p53 immunohistochemistry.^4^, ^19^ Two examples of *POLE*mut EC are illustrated in Figure 2. Figure 2A,B shows an EC case which was originally diagnosed as a stage II grade 3 EEC and Figure 2C,D shows an EC case that was diagnosed as a stage IB mixed endometrioid–clear cell EC. According to the current adjuvant treatment recommendations, both these cases are considered ‘high‐risk’ and adjuvant (external beam) radiotherapy is recommended and sequential chemotherapy may be considered.^20^

### How May the Presence of a Pathogenic *POLE* Mutation Affect Adjuvant Treatment Recommendations?

Despite their association with high‐grade histology, *POLE*mut EC patients have an exceptionally good prognosis.^5^, ^21^, ^22^, ^23^ Most of the studies to date have analysed retrospective cohorts in which patients had received some form of adjuvant treatment, so hypersensitivity to these adjuvant treatments could, theoretically, be the basis for the favourable outcomes of *POLE*mut ECs in these series. Sub‐analysis of *POLE*mut and *POLE*wild‐type EC in the observation arm of the PORTEC‐1 trial (*n* = 245 patients with stage I intermediate risk EC), however, also showed a 10‐year recurrence‐free survival (RFS) of 100% versus 80.1%, respectively (hazard ratio = 0.143; 95% confidence interval = 0.001–0.996; *P* = 0.049).^24^ This finding suggests that *POLE*mut EC possess intrinsic characteristics that are beneficial for survival, independent of sensitivity to adjuvant treatment. This is supported by the lack of an increased radiation sensitivity in *POLE*mut embryonic stem cells.^25^ It has been suggested that *POLE*mut EC are immunogenic due to their high mutational load, which may be a more plausible explanation for the unusually low chance of recurrences observed in these patients.^26^ Importantly, in addition to the excellent prognosis of intermediate‐risk *POLE*mut EC, similar survival rates have now been reported for patients with high‐risk *POLE*mut EC.^21^, ^22^, ^23^, ^27^ This consistent finding of excellent clinical outcomes in *POLE*mut ECs raises the question of whether these patients may be unnecessarily exposed to unwanted side‐effects of radio‐ and/or chemotherapy.^28^ Treatment de‐escalation towards perhaps no additional treatment at all for patients with *POLE*mut EC has therefore been proposed.^25^ The results of the ongoing PORTEC4a will show if this is a valid approach for patients with intermediate‐risk *POLE*mut EC. For high‐risk *POLE*mut EC, the recently presented molecular characterisation of the PORTEC‐3 trial has been highly informative.^27^ Patients with *POLE*mut EC showed an excellent prognosis independent of the treatment arm (EBRT versus CTRT), supporting omitting the addition of chemotherapy for patients with high‐risk *POLE*mut EC.

### Does the Co‐occurrence of *TP53* Mutations Affect the Management of *POLE*mut EC?

The majority of EC can be classified into one of the four molecular subgroups. However, in a small subset (3–5%) of patients molecular analysis will show more than one classifying alteration (e.g. *POLE*mut‐MMRd EC, *POLE*mut‐p53abn EC, MMRd‐p53abn EC or *POLE*mut‐MMRd‐p53abn EC), also referred to as ‘multiple classifier’ EC.^19^ As there are distinct prognostic differences between the four molecular subgroups, the question arises as to which biological behaviour these multiple classifiers follow. This dilemma is most pronounced in the combination of *POLE*mut‐p53abn EC, in which the tumour exhibits both a favourable pathogenic mutation in the *POLE* exonuclease domain as well as unfavourable aberrant p53 IHC expression, such as illustrated in our case in Figure 2A,B.

In contrast to the excellent prognosis of *POLE*mut EC, p53abn EC are associated with poor clinical outcomes.^5^, ^21^, ^23^ For some time, it has been uncertain how to classify EC in which a pathogenic *POLE* variation and a *TP53* mutation co‐occur. Molecular clustering of these ‘multiple classifier EC’ showed that *POLE*mut‐p53abn EC clustered together with *POLE*mut EC without *TP53* alterations, and it was noted that p53‐IHC in these cases frequently showed ‘subclonal’ mutant‐like p53 expression.^19^ Subclonal expression was defined as abrupt and complete regional aberrant p53 expression, in which the subclonal region was at least 10% of the total tumour volume. This unusual p53 expression pattern can be observed in *POLE*mut and MMRd EC, and reflects their genetic heterogeneity. Available survival data demonstrated that *POLE*mut‐p53abn EC show clinical outcomes comparable to *POLE*mut EC without abnormal p53 expression. These findings show that *TP53* mutations in these ‘multiple‐classifiers’ are probably passenger mutations not affecting the clinical behaviour, indicating that these cases should be classified and treated as *POLE*mut EC.

## MMRd endometrioid endometrial cancer

**Figure 3:**
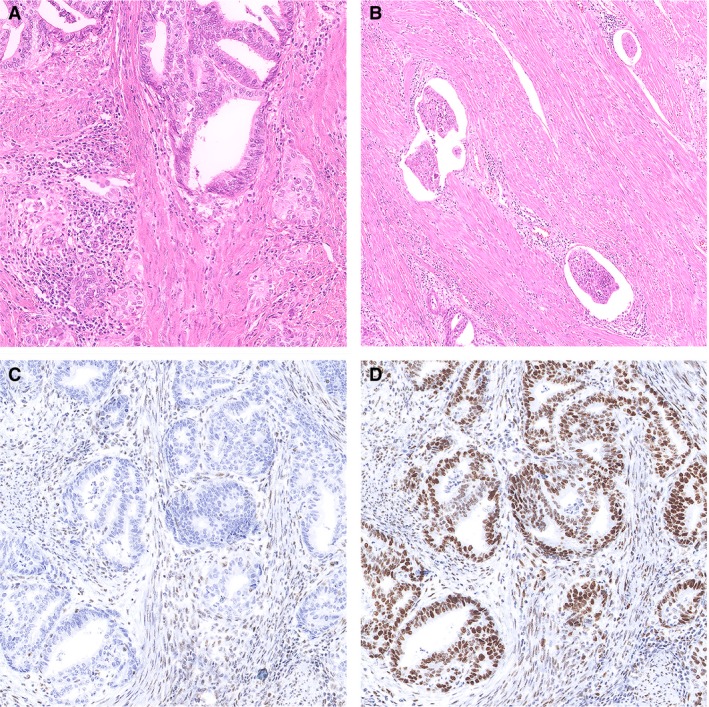
Example of a mismatch repair‐deficient (MMRd) endometrioid endometrial cancer (EEC) with lymphovascular space invasion (LVSI). **A**, Haematoxylin and eosin (H&E) stain of an endometrioid EC (FIGO grade 1), with prominent peritumoural lymphocytes. **B**, Representative image of the invasive front with foci of LVSI; the tumour contained >5 foci and was thereby reported as EC with substantial LVSI. **C**,**D**, Absence of nuclear immunoreactivity of PMS2 (**C**) and retained nuclear staining of MSH6 (**D**).

MMR deficiency is frequent in EC (25–30%) and defined by the loss of nuclear expression of one or more MMR proteins (MLH1, PMS2, MSH6 and PMS2) by the tumour cells.^4^, ^5^, ^21^, ^23^, ^29^ Reflex testing for MMR status is increasingly recommended by (inter)national guidelines for the identification of patients (and families) who are at high risk of having Lynch syndrome.^30^ The vast majority of MMR deficiency identified by this approach, however, is due to sporadic causes (promotor hypermethylation of *MLH1* or somatic MMR gene mutations), unrelated to Lynch syndrome.^31^ Histologically, MMRd EC show similarities to *POLE*mut EC in that these are also associated with higher‐grade, endometrioid‐type histology and abundance of tumour‐infiltrating lymphocytes (TILs).^4^, ^32^, ^33^ MMRd EC, however, have an intermediate prognosis, which significantly differs from *POLE*mut EC.^27^ Long‐term analysis of the PORTEC‐2 trial – comparing adjuvant VBT with EBRT in high–intermediate risk (HIR) EC – found that VBT was equally effective in preventing pelvic lymph node recurrences in the absence of unfavourable risk factors (p53abn, L1CAM, substantial LVSI), thus including HIR MMRd ECs having a very low absolute risk.^24^ In addition, for patients with HR MMRd EC, EBRT provides good pelvic control, and additional chemotherapy does not seem to improve prognosis.^27^ An example illustrated in Figure 3 depicts a case of a stage IB MMRd EEC with unequivocal lymphovascular space invasion (LVSI). According to the current adjuvant treatment recommendations, this patient is regarded as ‘high–intermediate risk’, and adjuvant radiotherapy is recommended.^20^

### How Does the Presence of (Substantial) LVSI Relate to MMRD EC?

Histologically, LVSI is defined as the presence of tumour cells within an endothelial‐lined space that lies outside the invasive border. Interestingly, identifying one focus of LVSI (also referred to as ‘focal’) has little impact on prognosis, whereas substantial LVSI (also referred to as ‘extensive’) is associated with a significant increase in the risk of recurrence.^34^ Substantial LVSI is seen in a small proportion of high–intermediate risk EC (~5%).^5^, ^24^, ^34^ The relevance of stratifying the extent of LVSI has only recently been accepted, and will soon be adopted in pathology reporting. Although substantial LVSI has been described in all molecular EC groups, an association between the presence of substantial LVSI and MMRd has been noted, with a reported prevalence of substantial LVSI in up to 8.9% of MMRd EC (*P* = 0.002).^5^, ^35^ This association may explain the fact that a relatively large proportion of high‐stage EC fall within the MMRd subgroup.^36^ How MMRd leads to LVSI in EC is not well understood, but it may be the result of frequent epithelial‐to‐mesenchymal transition in MMRd EC.^37^

### How Does the Presence of Substantial LVSI Impact Adjuvant Treatment in EC?

Multiple studies investigating the prognostic impact of (substantial) LVSI in EC patients have found an increased risk of lymph node metastasis and locoregional as well as distant recurrences, regardless of stage and histotype.^34^, ^38^, ^39^, ^40^, ^41^, ^42^ These studies suggest that adjuvant radio‐ or chemotherapy is recommended to reduce the risk of recurrence in these patients. This is supported by the long‐term results of the PORTEC‐2 trial – including patients with high–intermediate risk factors – that found a decreased risk of pelvic lymph node recurrence after EBRT compared to VBT and postoperative observation for HIR EC patients with substantial LVSI.^24^ Importantly, although LVSI is seen mainly in MMRd EC, LVSI is also an independent prognostic factor in NSMP and p53mut EC. Therefore, LVSI assessment will remain essential in risk stratification schemes, escalating adjuvant treatment regimens in those patients in whom substantial LVSI is identified. Given the lack of surrogate markers, pathologists will continue to be asked to provide this information based on H&E assessment, which has an acceptable level of interobserver agreement.^43^ As substantial LVSI is such a strong prognostic factor, LVSI illustrates that histological characteristics need to be integrated with the molecular classification to reach an optimal risk stratification in EC.

## p53 Mutant low‐grade endometrioid endometrial carcinoma

**Figure 4:**
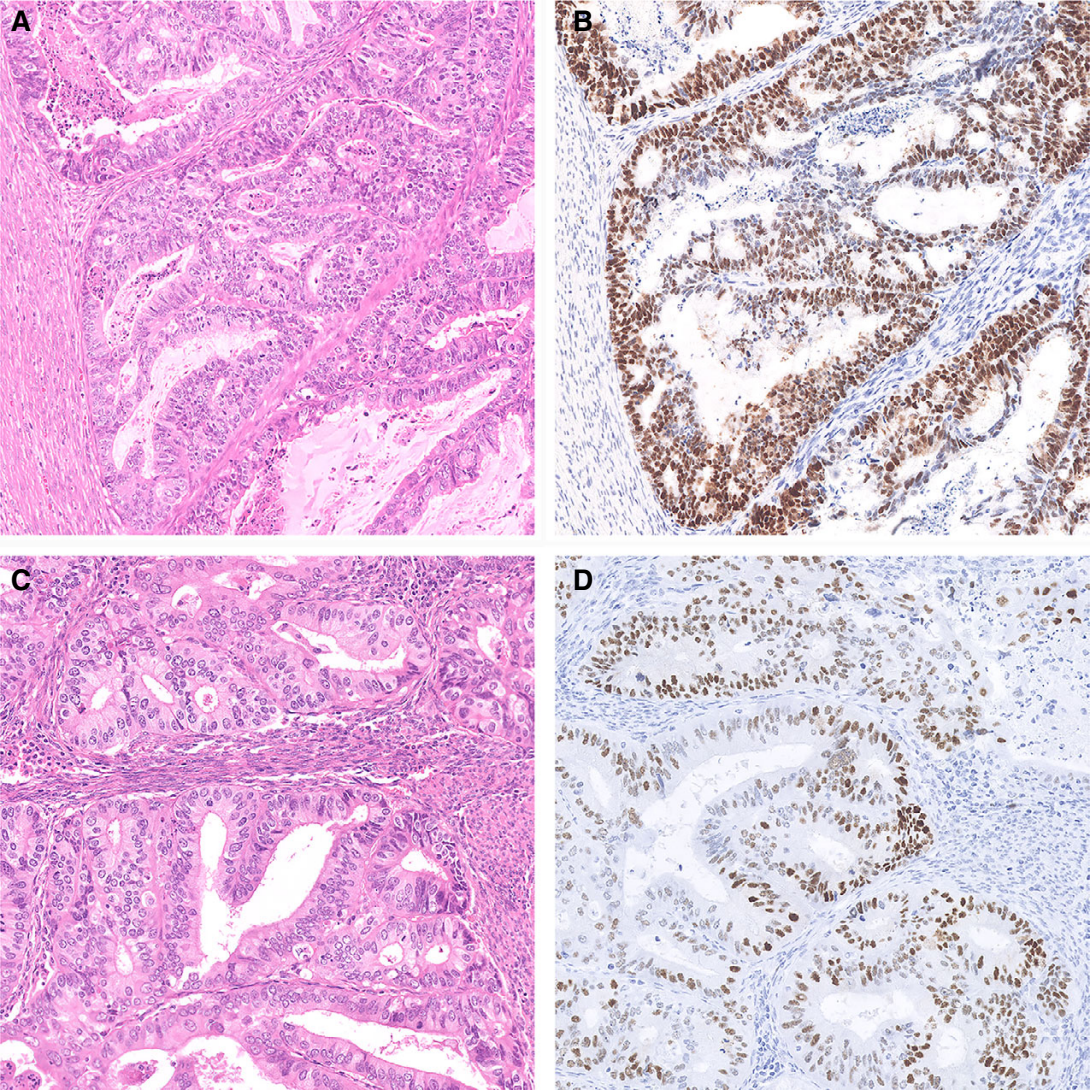
Two examples of low‐grade p53 mutation endometrioid endometrial cancer (EC). **A**, Representative haematoxylin and eosin (H&E) stain of an EC diagnosed as FIGO grade 2 (based on nuclear atypia) endometrioid EC. **B**, This case showed diffuse nuclear overexpression of p53 by IHC, interpreted as mutant‐like expression. Next‐generation sequencing (NGS) confirmed the presence of a *TP53* mutation. **C**, Another example of an EC diagnosed as FIGO grade 1 endometrioid EC with aberrant p53 staining. NGS confirmed the presence of a pathogenic *TP53* mutation. Both cases were mismatch repair protein (MMR)‐proficient and did not carry a polymerase‐epsilon (*POLE*) mutation.

A paradoxical scenario occurs when a morphological low‐grade endometrioid EC shows molecular characteristics associated with aggressive clinical behaviour, such as abnormal mutant‐type p53 staining. This is a rare finding in low‐grade EEC, as it is reported in 2–15% of glandular EEC with low nuclear grade,^5^, ^6^, ^21^, ^29^, ^44^, ^45^ whereas it is a more common finding (10–15%) in high‐grade EECs.^36^ Two examples of low‐grade EEC with *TP53* mutations and abnormal p53‐IHC are shown in Figure 4. Both these tumours were diffusely positive for hormone receptors and showed loss of PTEN immunostaining, supporting the ‘endometrioid’ classification. Although the nuclear atypia in these tumours may alarm some pathologists for a ‘glandular variant’ of serous endometrial cancer, tumours such as this with smooth luminal borders continue to be difficult to distinguish from low‐grade EEC.^46^, ^47^ Both these cases were originally reported as a stage IB, low‐grade EEC and p53 was not performed. According to the current adjuvant treatment recommendations, both patients would be regarded as ‘intermediate‐risk’ and adjuvant vaginal brachytherapy is recommended.^20^

### How Does the Presence of a *TP53* Mutation Impact the Adjuvant Treatment in Endometrial Cancer?

p53 IHC has proved to be a very reliable surrogate marker for detecting underlying *TP53* mutations in EC, with reported sensitivity and specificity of 0.96 and 1.00, respectively.^48^, ^49^, ^50^ Importantly, several studies have shown that patients with p53mut EC, independent of histotype, grade or stage, show poor clinical outcomes.^5^, ^21^, ^23^, ^29^, ^45^ The number of low‐grade EC which fall into the p53mut EC subgroup is limited, but the available data point towards unfavourable clinical outcomes in these cases.^5^, ^24^, ^45^ It has been suggested that combining p53 IHC with the classical histological grading system will improve prognostic accuracy for EEC.^45^ However, we would prefer to see p53mut EC as a separate entity within the integrated histomolecular EC classification. The reported poor clinical outcomes have been the rationale to propose adjuvant treatment escalation from VBT to EBRT in the PORTEC‐4a trial for high–intermediate‐risk EC patients in the uncommon scenario of mutant‐like p53 immunostaining in these patients. For high‐risk patients with a p53mut EC, such as those enrolled in the PORTEC‐3 trial, further treatment escalation by additional chemotherapy significantly improves clinical outcomes (5‐year RFS with CTRT 61 versus 37% for RT).^27^

## Potential role for molecular characteristics in the treatment of recurrent or advanced EC

To date, treatment options for patients with advanced or recurrent EC are limited. In recent years several targetable pathways in the context of molecular EC subgroups have been investigated, resulting in novel treatment strategies with potential clinical benefit. *POLE*mut and MMRd EC, due to their high mutational burden, obtain high levels of neoantigens and TILs, making them attractive candidates for immunotherapy such as anti‐PD1 immune check‐point blockade.^32^, ^33^, ^51^, ^52^, ^53^, ^54^, ^55^, ^56^, ^57^ Despite this theoretical argument, the excellent clinical outcomes for patients with *POLE*mut EC under current treatment regimens (independent of stage), however, argues against the rationale to the use of immunotherapy for this subpopulation. For MMRd EC, particularly when recurrent or in advanced metastatic stage (FIGO stage >III), immune check‐point inhibition may be an attractive option.^53^ Currently, there is no good rationale for the use of check‐point inhibition in the context of p53mut EC. As patients with p53mut EC represent the group with the worst clinical outcomes, identifying targets in p53mut EC is an urgent need. Targeting amplifications of the *ERBB2* gene, encoding for human epidermal growth receptor 2 (HER2) and homologous recombination deficiency (HRD), both occurring in p53mut EC, are being explored and show some promise.^58^

### Role of HER2 Expression in EC Treatment

**Figure 5:**
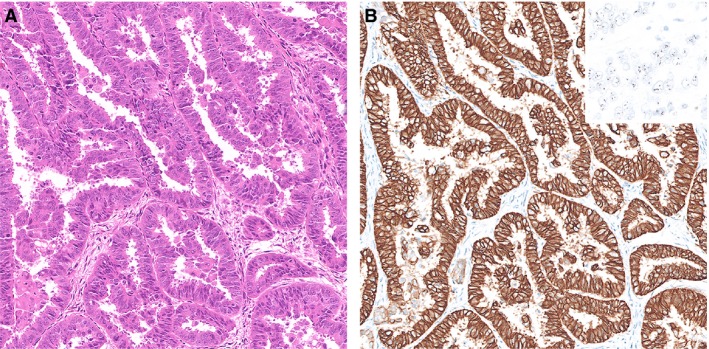
Example of human epidermal growth factor receptor 2 (HER2) overexpressing serous endometrial cancer (EC). **A**, Representative haematoxylin and eosin (H&E) stain of a serous EC with profound nuclear atypia and presence of a rugged luminal surface. **B**, Complete strong membranous HER2 immunohistochemical staining. In this case the *ERBB2* amplification was confirmed with *in‐situ* hybridisation (see inset).

Amplification of the *ERBB2* gene and/or HER2 overexpression have been reported in serous EC and uterine carcinosarcomas with a serous carcinomatous component, albeit with a substantial variation in reported rates of 14–80% and 21–47%, respectively.^59^, ^60^ This variation can be explained by the lack of standardised scoring methods for HER2 immunohistochemistry in EC. Most studies have scored HER2 IHC expression using scoring guidelines designed for breast cancer; however, HER2 expression shows significant heterogeneity in EC, in contrast to HER2‐positive breast cancer, and therefore these guidelines may not be directly applicable to EC.^61^ An example of a serous EC with diffuse immunohistochemical overexpression of HER2 and amplification of *ERBB2* by *in‐situ* hybridisation is shown in Figure 5. Trastuzumab is a monoclonal antibody directed against the HER2 receptor and, when combined with chemotherapy, has been shown to increase survival in HER2 overexpressing breast and gastric cancer.^62^, ^63^ Studies investigating the therapeutic potential of trastuzumab as a single agent in HER2 overexpressing EC were not able to demonstrate any prognostic benefit.^64^, ^65^ However, a recent trial including patients with stages III/IV or recurrent HER2 overexpressing serous EC found an increased progression‐free survival (PFS) from 8 to 13 months (*P* = 0.005) when trastuzumab was given in combination with carboplatin‐paclitaxel chemotherapy.^58^ These findings encourage further investigation on the efficacy of trastuzumab combined with chemotherapy to improve outcomes for patients with advanced or recurrent EC. For pathologists, the strong association between *ERBB2* gene amplifications and serous histology is of interest, as it suggests that this genomic alteration may be limited to p53mut EC. It would be informative to address this association in more detail in future studies, as it would provide a rationale for focused HER2 testing in the context of p53mut EC.

### Role of HRD in EC Treatment

Homologous recombination repair capacity has been successfully used as a marker to identify patients that benefit from poly (ADP ribose) polymerase inhibitors (PARPi) in high‐grade serous ovarian cancer (HGSOC).^66^, ^67^ HGSOC and serous EC have very similar molecular genetic alterations^4^; recent data show that a subset of p53mut EC are homologous recombination‐deficient, and some of these EC can arise in the context of germline *BRCA1/2* mutations.^68^, ^69^, ^70^, ^71^ The exact prevalence of HRD in p53mut EC is currently unknown; in a small and selected set of cases it was 46%.^68^ Together, these data build upon a rationale to target homologous recombination in p53mut EC, particularly those that are HRD. Currently, several clinical trials investigating the efficacy of different PARP‐inhibitors in recurrent or metastatic EC are either planned or recruiting; the results of these trials are eagerly awaited. In the meantime, the gold standard for testing the homologous recombination status of tumours, including EC, should be defined.

## Discussion

The molecular subgrouping proposed by the TCGA has been fundamental in evolving the dualistic Bokhman model into a more refined and molecular‐based system. The subsequent development and validation of widely accessible surrogate markers for the four distinct EC subgroups has accelerated this field. This has led to a diagnostic algorithm that incorporated molecular characteristics, resulting in a novel, objective and clinically meaningful EC classification. Once regulatory authorities understand the potential clinical impact of this improved EC classification, financial aspects that come with the implementation of additional testing should be quickly resolved. Pathologists will be asked to assess the mutational status of the *POLE* gene and perform three immunohistochemical stains (p53, MSH6 and PMS2) in order to provide an integrated ‘histomolecular’ diagnosis. Next, the design of (adjuvant) treatment strategies incorporating this novel classification will become a priority. This shift towards a molecular driven EC classification is yet another illustration of how pathology is delivering the promise of precision medicine.

The molecular EC classification follows the subclassification proposed by the TCGA; however, further refinement of this classification is to be expected. It would be particularly attractive to further refine the largest EC subgroup, NSMP EC, which can be regarded as a heterogeneous molecular rest group mainly consisting of alterations in PI3K‐Akt and Wnt‐signalling alterations.^4^ NSMP EC are almost exclusively low‐grade endometrioid‐type endometrial carcinomas. One molecular alteration that stands out in terms of frequency is the presence of mutations in exon 3 of *CTNNB1* (52%).^4^ Subsequent studies evaluating the potential prognostic significance of these *CTNNB1* mutations in low‐grade early‐stage EC showed a significantly worse recurrence‐free survival.^72^, ^73^ In addition, independent from the TCGA data, *CTNNB1* exon 3 mutations were shown to have prognostic significance in patients with high–intermediate‐risk NSMP EC.^5^ Morphologically, *CTNNB1* exon 3‐mutated EC are associated with low‐grade, low abundance of TILS and with squamous differentiation.^72^, ^74^ Studies investigating both β‐catenin IHC and *CTNNB1* exon 3 gene sequencing in EC have reported varying concordance rates, resulting in the conclusion that β‐catenin IHC is insufficient to act as a surrogate marker for *CTNNB1*mut EC.^72^, ^74^, ^75^ The validated prognostic impact within NSMP EC, in combination with the distinct morphological features, may qualify *CTNNB1* mutant EC as the fifth molecular EC subgroup, which may be referred to as *CTNNB1*mut EC (after excluding MMRd and *POLE* mutations). It is conceivable that *CTNNB1*mut EC will be incorporated into the diagnostic algorithm as a separate ‘histomolecular’ entity.

Most studies that report on the impact of adjuvant treatment in the context of the molecular EC classification have included high‐intermediate risk patients. For patients who would currently be regarded as ‘low‐’ or ‘high‐risk’, the current data on the impact of adjuvant treatment are limited. Stelloo *et al*. showed, in an univariable analysis of 242 EEC with low‐risk features, a trend towards a higher rate of distant recurrences and lower overall survival (*P* = 0.061 and *P* = 0.058, respectively) for patients with p53mut EC.^5^ Despite this impact on prognosis, these data are insufficient to support adjuvant treatment recommendations based on molecular classification of low‐risk EC, and further research is needed. In addition, it would be interesting to study the effect of fertility‐sparing progestin therapy among the four molecular subgroups, as this can be informative for the management of low‐risk EC in young women of reproductive age. In contrast to low‐risk EC, it is becoming very clear that in high‐risk EC molecular subgroups not only impact prognosis, but should also influence adjuvant treatment decisions. This is exemplified by *POLE*mut EC in the high‐risk EC population, which show an excellent prognosis when adjuvant treatment is limited to radiotherapy, strongly suggesting that these patients do not benefit from adjuvant chemotherapy.^27^

This review shows that changing the EC classification to include molecular characteristics provides exciting opportunities, but will also create certain challenges with respect to testing and reporting. These challenges include the availability and choice of assays for surrogate markers, as well as the management of patients in centres that do not have access to these assays. Immunohistochemical surrogate markers, such as p53 and MMR‐protein immunohistochemistry, are readily available, but it is recognised that some centres may not have the ability to carry out *POLE* testing or that results from any of the required assays may not be conclusive. To molecularly classify an individual EC, following the diagnostic algorithm provided in Figure 1, *POLE* testing is a prerequisite. This is best illustrated by *POLE*mut EC that carry *TP53* mutations, which would be falsely classified as p53mut EC when *POLE*‐testing is not performed. With this in mind, we propose that a ‘not otherwise specified’ (NOS) diagnostic designation is used when there is insufficient information to assign a specific molecular entity. This is in contrast to ‘NSMP EC’, which defines EC that have been completely profiled, but do not carry a specific classifying feature. Our proposed ‘NOS’ designation is used in combination with a specified histological subtype (e.g. EEC‐NOS, SEC‐NOS, CCC‐NOS) to ensure that the information of histological subtyping is not lost in these cases. We believe that the introduction of this NOS designation will provide necessary clarity in translational research and in reporting to clinicians.

## Conclusion

The scenarios presented illustrate that the molecular EC classification provides clinically relevant and prognostic information, with the potential to influence (adjuvant) treatment strategies. When clinical trials confirm the added value of integrated molecular diagnostic entities, we envision that this will become the new standard in EC reporting. Independent of ‘histomolecular’ type, certain histopathological characteristics, such as (the extent of) LVSI and stage, do not have a molecular surrogate and will remain essential in the pathological assessment of a hysterectomy with EC. The value of histological type and FIGO grade is less certain, but it is recommended to still report on these in the years to come. The precise weight of all these intrinsic tumour variables should be evaluated to design a novel risk stratification scheme for adjuvant treatment that includes some of the most relevant molecular characteristics. Finally, the molecular classification provides a framework for subclass‐specific trial designs that should be developed over the coming years to examine the potential benefit of subclass‐specific targeted therapy.

## Conflict of interest

All authors declare no conflicts of interest.
